# Acquired Urethral Diverticulum After Holmium Laser Enucleation of the Prostate: A Case Report

**DOI:** 10.7759/cureus.57068

**Published:** 2024-03-27

**Authors:** Ryan R Chen, Joao G Porto, Ruben Blachman-Braun, Ramgopal K Satyanarayana, Hemendra N Shah

**Affiliations:** 1 Urology, Desai Sethi Urology Institute, University of Miami, Miami, USA

**Keywords:** case report, benign prostatic hyperplasia, urinary incontinence, holmium laser enucleation, urethral diverticulum

## Abstract

Acquired urethral diverticula (UD) in males is an uncommon entity, and it is rarely reported after an open simple prostatectomy or transurethral resection of the prostate. Here, we report a unique case of a UD presenting after holmium laser enucleation of the prostate (HoLEP) in a 69-year-old male with a prostate of 372 g who had five episodes of urine retention over one year despite combined medical treatment with tamsulosin 0.8 mg and finasteride 5 mg. The patient also has elevated prostate-specific antigen (PSA) with five negative prostate biopsies over the last few years. The procedure lasted six hours with difficult morcellation due to beach balls that took 3.5 hours. There were no intraoperative complications. However, he continued to have mixed urine incontinence and recurrent (six) episodes of urinary tract infection (UTI) in the first postoperative year. On evaluation, his urodynamic study did not reproduce stress urinary incontinence (SUI); however, cystoscopy and retrograde urethrogram diagnosed a 6-cm UD in the bulbar penile urethra with penoscrotal mass. The patient underwent urethral diverticulectomy and urethroplasty with a buccal mucosa graft to correct the defect. Six months after his urethral reconstruction, he continued to have mixed urine incontinence needing two pads/day. Although male UD is a rare condition, our case report seeks to heighten awareness of such a potential rare complication in men with recurrent UTIs and refractory urinary incontinence after prolonged HoLEP for extremely large prostates.

## Introduction

Urethral diverticula (UD) manifest as saccular dilations contiguous with the true urethral lumen. Although more prevalent in women due to weaker anatomical support, they are seldom observed in men [1,2]. Acquired factors, including strictures, infection, trauma, indwelling urethral catheters, or prior surgeries, contribute to about 90% of cases [3-5].

Benign prostatic hyperplasia (BPH) primarily affects older males, with transurethral resection of the prostate being the conventional surgical intervention. There are very few cases of acquired UD reported in the literature following surgical management of an enlarged prostate either following open simple prostatectomy or transurethral resection of the prostate [1]. Over the past decade, holmium laser enucleation of the prostate (HoLEP) has gained popularity due to its efficacy across different prostate sizes [6]. This report presents, to the best of our knowledge, the first case in the literature detailing a UD following HoLEP.

## Case presentation

**Figure 1:**
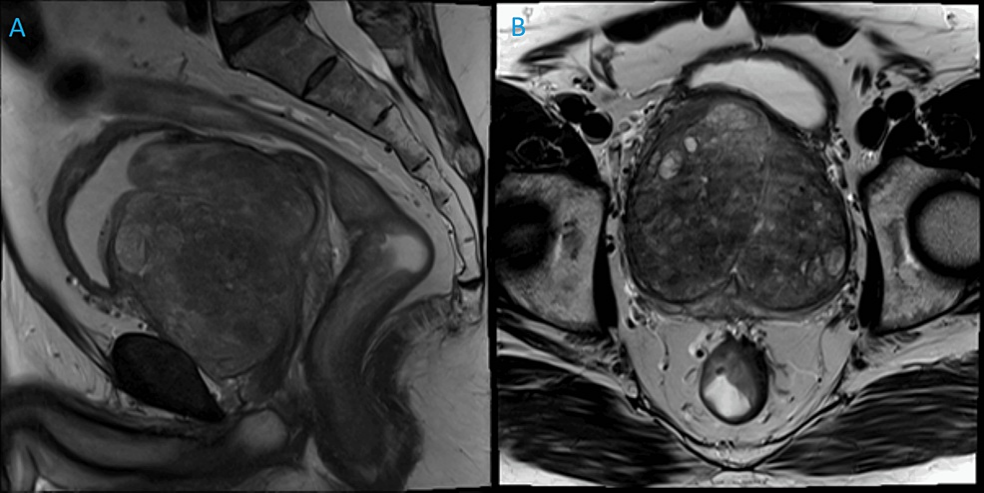
Preoperative prostate MRI showing gland of 372 g with findings of prostatic hyperplasia within the transition zone and no suspicious lesions of prostate cancer. (A) Sagittal prostate MRI. (B) Coronal prostate MRI.

A 69-year-old male presented to our office with bothersome lower urinary tract symptoms for one year despite taking tamsulosin 0.8 mg and finasteride 5 mg over the last two years. His International Prostate Symptom Score was 16 points. He denied urinary incontinence and had five previous episodes of acute urinary retention (AUR) needing catheter placement for one to four weeks on each occasion. He had a history of elevated prostate-specific antigen (PSA) levels, with the most recent reaching 15.74 ng/ml. He had five negative prostate biopsies over the last few years and his 4K score was 3%. His multiparametric prostate magnetic resonance imaging (MRI) revealed a 372.87 g prostate with no suspicious lesions of cancer (Figure 1). Various surgical options were discussed with the patient such as open or robotic simple prostatectomy, and he consented to HoLEP. The procedure was performed under general anesthesia using 550-micron holmium laser fiber (Lumenis Ltd, Israel) at the setting of 2 J and 30 Hz using the en-bloc technique.

During the initial evaluation of the urethra and cystoscopy, no outpouching or other abnormalities were detected. Adenoma was morcellated using 26 Fr. Nephroscope (Karl Storz Endoscopy, Tuttlingen, Germany) and Lumenis VersaCut morcellator. The morcellation was difficult due to large beach balls and took 3.5 hours. Many small-sized beach balls were removed transurethrally with a Perc-NCircle basket (Cook Medical LLC, Bloomington, IN) using a nephroscope. The procedure lasted six hours in total without any intraoperative complications. Hemoglobin dropped from 14.2 to 10.3 g/dl on the first postoperative day. The patient was discharged the next day after a successful voiding trial with a recommendation to perform Kegel exercises for the improvement of transient urinary incontinence. The subsequent histopathological examination of resected prostate tissue revealed 353.2 g of BPH tissue.

At his six-week follow-up visit, he complained of dysuria and mixed urinary incontinence (MUI), requiring four to five pads/day. Specifically, he experienced involuntary leakage of urine associated with physical exertion, such as coughing or exercising. Additionally, he reported a sudden and intense urge to urinate that was difficult to control, leading to involuntary urine leakage before he could reach the bathroom. The patient was recommended to continue Kegel exercises and was given a trial of oxybutynin 10 mg. At three months of follow-up, the patient reported having a strong urinary stream with complete emptying of the bladder. However, he reported having MUI with the use of three to four pads/day but denied the use of oxybutynin and followed behavioral recommendations. At that time, the need to use medication and perform Kegel exercises was reinforced. His PSA nadir at three after HoLEP was 0.48 ng/ml.

At six months, he reported that he had stopped taking oxybutynin due to xerostomia but stated to do Kegel exercises regularly with minimal improvement of urinary incontinence in the last three months. Mirabegron 25 mg and duloxetine 20 mg were prescribed, with a referral to physical therapy rehabilitation (12 sessions every two weeks) with a recommendation to continue pelvic floor exercises at home. This led to a subjective 20% improvement in incontinence nine months after HoLEP, but he was still using two pads/day. At the 11-month postoperative period, he informed that he started passing per-urethral tissue with no pain, hematuria, or dysuria. A subsequent renal ultrasound revealed layering of debris in the patient’s bladder, and a urodynamic study (UDS) could not demonstrate any stress urinary incontinence (SUI). A cystoscopy showed a large UD (100 ml) with an opening in the penile-bulbar urethral junction. The same was palpable in the penoscrotal junction with a size of approximately 6 cm, and a significant amount of malodorous urine could be expressed upon manual pressure. In the entire first year after HoLEP, he had six episodes of culture-positive UTI needing antibiotics.

**Figure 2:**
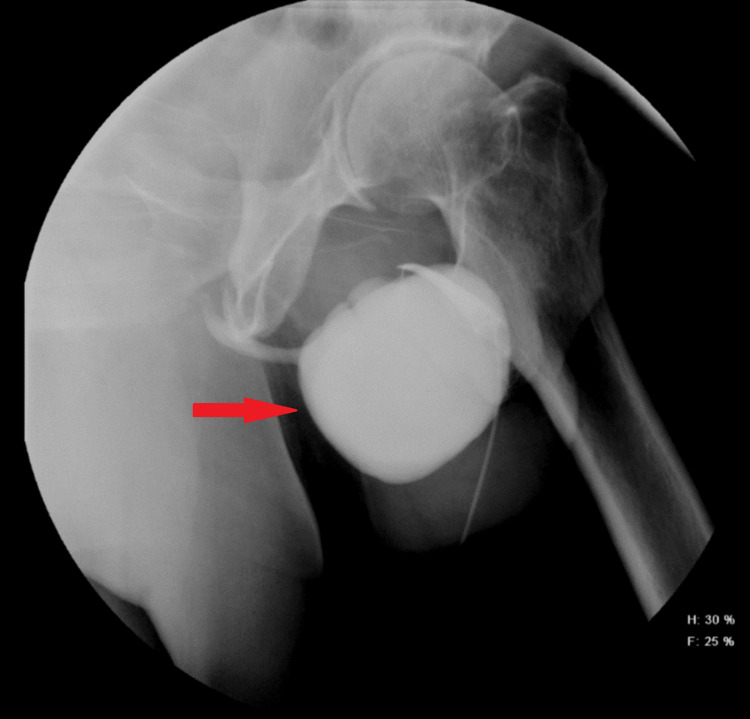
Retrograde urethrogram showing large urethral diverticulum filled with radiopaque contrast in the inferior basal aspect of the distal penile urethra, approximately 3.4 cm from the tip of the penis measuring 6.6 x 5.0 x 5.4 cm

The patient was referred to a reconstructive urologist who conducted a retrograde urethrogram (RUG), confirming the diagnosis (Figure 2). The significant size of the defect coupled with recurrent UTIs led the patient to opt for a surgical intervention. During the preoperative counseling, no SUI was evident following manual decompression of the UD; however, the patient reported experiencing SUI at other times. Subsequently, he underwent a penoscrotal incision for a urethral diverticulectomy with primary urethroplasty and buccal mucosal grafting. A large UD measuring approximately 10 x 8 cm with a volume of 100 ml was identified during the procedure. No intraoperative complications occurred, and the patient was discharged on the first-day post-surgery with a Foley catheter in place.

**Figure 3:**
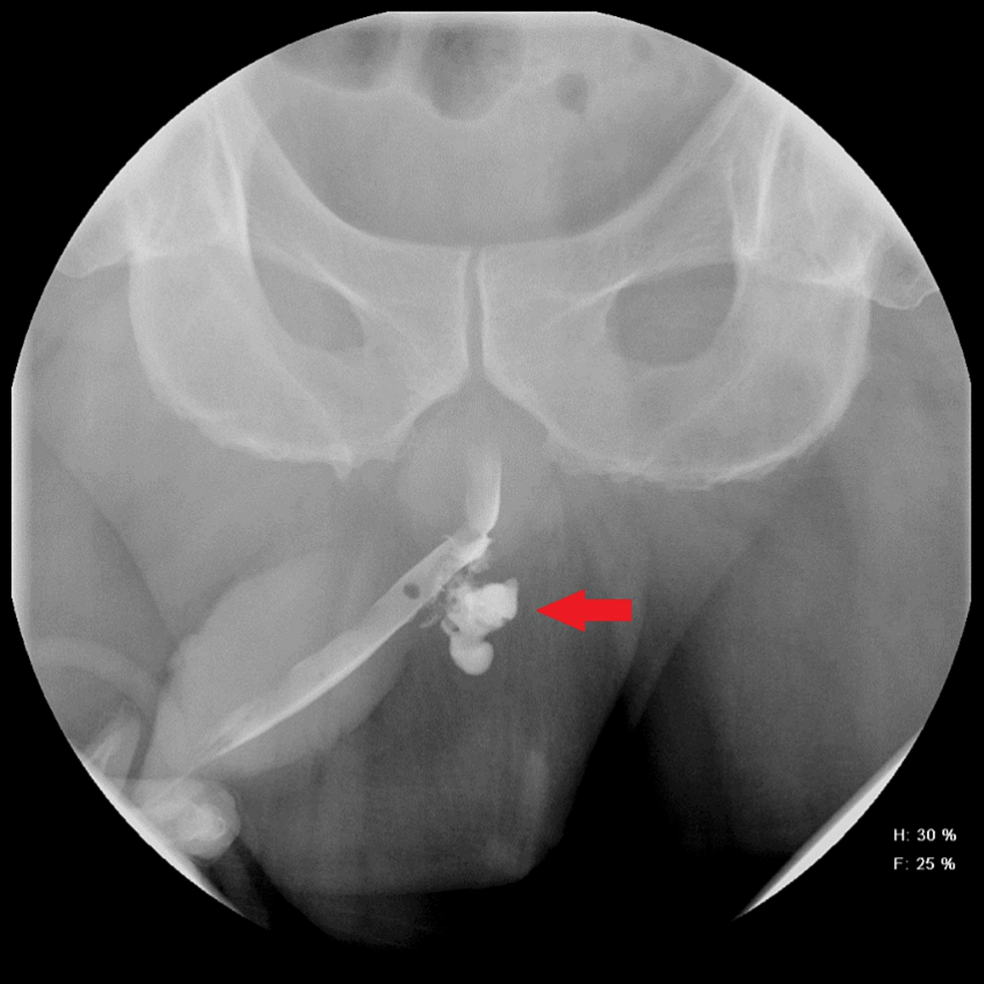
Retrograde urethrogram showing mild leakage of contrast posteriorly from the proximal bulbous urethra

A RUG was done 10 days later showing mild leakage in the proximal bulbous urethra (Figure 3), and the Foley catheter was removed. Three months after urethroplasty, the patient denied any new episode of UTI, and cystoscopy showed a well-healed urethra with no strictures. Uroflowmetry revealed a peak flow of 35.3 ml/s and a postvoid residual volume of 8 ml. However, at the six-month post-urethral reconstruction, the patient continues to have SUI using two pads/day although he has not been leaking as much as before urethroplasty. After 18 months post-HoLEP, he is inclined to consider definitive surgical intervention for his incontinence in the near future.

## Discussion

Acquired male UD is known to be a complication of prostatic and anorectal procedures, especially in patients with indwelling catheters for extensive periods [7-10]. These procedures include recto-urethral fistula repair, single-stage urethroplasty, hypospadias correction, artificial urinary sphincter placement, and transurethral bladder/prostate procedures [1,8,9,11,12]. Clinical presentation often includes obstructive urinary symptoms, postmicturition dribble/urinary incontinence, recurrent UTIs, penoscrotal mass, and urinary retention [1,12]. Our patient had similarly experienced recurrent UTIs, prolonged urinary incontinence, and penoscrotal fullness. However, he presented with a strong stream and passing per-urethral tissue, which has not been noted before in the literature.

Historically, the prevailing notion attributed male-acquired UD to potential nerve pathway damage resulting from pressure necrosis [13]. However, contemporary understanding emphasizes that the underlying mechanism depends on the specific cause behind this condition [1]. In instances where patients have chronic indwelling catheters, the constant pressure exerted on the penoscrotal angle can trigger urethral ischemia, subsequently leading to fibrosis [14]. For those with a complex urological history involving prior reconstructive surgeries, there is a risk of urethral obstruction formation that might lead to subsequent epithelium herniation [12]. Additionally, anorectal malformation stands as another significant factor [8,15]. In the present report, while the patient had previous episodes of AUR requiring Foley catheter insertion, there is no record of challenging catheterizations or prolonged Foley catheter usage (beyond a few weeks). More importantly, during the initial urethral evaluation and cystoscopy, the patient exhibited no signs of diverticulum. Our hypothesis centers on the extended duration of the HoLEP with the prolonged presence of instruments causing ischemia at the penoscrotal junction that led to the formation of a diverticulum [9,11].

The acquired UD can be effectively managed through nonoperative methods, involving manual decompression of the penoscrotal mass. Patients who can successfully empty the diverticulum and experience UTI prevention through prophylactic antibiotics are eligible for this approach. However, a urethral diverticulectomy is recommended for individuals facing recurrent UTIs despite antibiotic use, obstructive voiding, or the presence of UD-associated stones. Additionally, another alternative involves urinary diversion, particularly relevant for patients with a neurogenic bladder requiring clean intermittent catheterization and symptomatic UD [1,9,12]. Our patient presented with a 6 cm UD and recurrent UTIs despite medical treatment. As a result, a surgical excision with primary urethroplasty was performed to address the condition effectively.

The present study has inherent limitations of a case report, such as limited generalizability and the retrospective evaluation of events. While UDs have been documented by two case series as a rare complication of transurethral resection, our study stands as the first to address the occurrence of UD following HoLEP [1,9]. The recognition of this uncommon pathology becomes crucial, particularly in cases following prolonged transurethral enucleation of the prostate. It is prudent to underscore strategies aimed at reducing the intraoperative duration, thereby diminishing urethral pressure and manipulation, with the potential to mitigate the incidence of such complications. In instances involving notably enlarged prostates, alternatives such as morcellation through a suprapubic cystotomy or adenoma removal via cystotomy merit consideration. Factors that decrease the morcellation efficiency are the “beach ball effect” (small and fibrotic prostatic tissue fragments), prostatic calcification, and increased morcellation weight tissue [16]. Additionally, it is pertinent to consider disclosing information regarding the limited improvement of SUI after urethral diverticulectomy and urethroplasty, particularly if the patient's incontinence abates following manual decompression of the UD. Larger studies with control groups are necessary to investigate the potential association between HoLEP and UD occurrence. Further research should explore other contributing factors and refine surgical techniques to minimize the risk of UD formation after prostate interventions. Reporting similar cases would contribute to a better understanding of this rare complication.

## Conclusions

Male UD stands as a rare complication that should be considered in the setting of recurrent UTIs and refractory urinary incontinence after prolonged HoLEP procedures treating large prostates. While managing the diverticulum is achievable through urethral diverticulectomy and urethroplasty, it is essential to emphasize that persistent SUI after successful manual emptying of the diverticulum might indicate ongoing incontinence post-surgery. Additionally, it is prudent to contemplate techniques for sidestepping transurethral adenoma extraction, potentially reducing the risk of UD occurrence after these procedures.
